# Synthesis, Conformation, and Activity of Human Insulin-Like Peptide 5 (INSL5)

**DOI:** 10.1002/cbic.200800113

**Published:** 2008-06-24

**Authors:** Mohammed Akhter Hossain, Ross A D Bathgate, Chze K Kong, Fazel Shabanpoor, Suode Zhang, Linda M Haugaard-Jönsson, K Johan Rosengren, Geoffrey W Tregear, John D Wade

**Affiliations:** a Howard Florey Institute, University of MelbourneVictoria 3010 (Australia), Fax: (+61)39348-1707 E-mail: john.wade@florey.edu.au; b Department of Biochemistry and Molecular Biology, University of MelbourneVictoria 3010 (Australia); c School of Chemistry, University of MelbourneVictoria 3010 (Australia); d School of Pure and Applied Natural Sciences, University of Kalmar391 82 Kalmar (Sweden)

**Keywords:** circular dichroism, insulin-like peptides, peptides, RXFP4, synthesis

## Abstract

Insulin-like peptide 5 (INSL5) was first identified through searches of the expressed sequence tags (EST) databases. Primary sequence analysis showed it to be a prepropeptide that was predicted to be processed in vivo to yield a two-chain sequence (A and B) that contained the insulin-like disulfide cross-links. The high affinity interaction between INSL5 and the receptor RXFP4 (GPCR142) coupled with their apparent coevolution and partially overlapping tissue expression patterns strongly suggest that INSL5 is an endogenous ligand for RXFP4. Given that the primary function of the INSL5–RXFP4 pair remains unknown, an effective means of producing sufficient quantities of this peptide and its analogues is needed to systematically investigate its structural and biological properties. A combination of solid-phase peptide synthesis methods together with regioselective disulfide bond formation were used to obtain INSL5. Both chains were unusually resistant to standard synthesis protocols and required highly optimized conditions for their acquisition. In particular, the use of a strong tertiary amidine, DBU, as *N*^α^-deprotection base was required for the successful assembly of the B chain; this highlights the need to consider incomplete deprotection rather than acylation as a cause of failed synthesis. Following sequential disulfide bond formation and chain combination, the resulting synthetic INSL5, which was obtained in good overall yield, was shown to possess a similar secondary structure to human relaxin-3 (H3 relaxin). The peptide was able to inhibit cAMP activity in SK-N-MC cells that expressed the human RXFP4 receptor with a similar activity to H3 relaxin. In contrast, it had no activity on the human RXFP3 receptor. Synthetic INSL5 demonstrates equivalent activity to the recombinant-derived peptide, and will be an important tool for the determination of its biological function.

## Introduction

**Figure 1:**
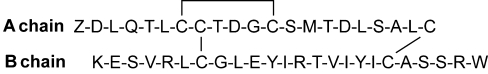
Predicted primary structure of INSL5.

Insulin-like peptide 5 (INSL5) was first identified through a search of the expressed sequence tags (EST) databases for novel insulin-like sequences.^1^ Primary structure analysis showed it to comprise of 135 amino acid residues with a signal peptide, B, that connected the C and A domains. It is postulated to be processed in vivo to yield a two-chain structure (A and B) that contains the insulin-like disulfide cross-links; this makes it a bona fide member of the insulin superfamily. The predicted primary structure of INSL5 consists of a twenty one residue A chain and a twenty four residue B chain that are linked by three disulfide bonds (Figure 1). Other members of the human insulin superfamily are insulin,^2^ IGF1,^3^ IGF2,^4^ relaxin-1, and relaxin-2 (H1 and H2 relaxin, respectively),^5^,^6^ INSL3,^7^ INSL4,^8^ INSL6,^9^ and relaxin-3 (H3 relaxin/INSL7).^10^ Northern blot analysis showed highest expression of human INSL5 in rectal and colon tissue;^1^ this suggests a probable role in gut contractility. Quantitative reverse transcriptase-polymerase chain reaction (RT-PCR) revealed the presence of INSL5 mRNA in a variety of human tissues, including the pituitary and, in lower levels, in the brain.^11^

In the mouse, the highest expression of INSL5 mRNA is in the colon^1^ and kidney.^12^ An INSL5 knockout mouse has been developed, which includes a *lacZ* reporter gene to track INSL5 expression at the cellular level.^13^ Although the phenotype of this mouse was not reported, *lacZ* expression was seen in a discrete population of cells in the colon. Although Northern blot analysis showed that INSL5 mRNA was absent in brain tissue or the pituitary,^1^ RT-PCR showed the presence of INSL5 mRNA in the hypothalamus.^14^ Immunohistochemical studies with an antiserum against the mouse INSL5 peptide showed the presence of INSL5-immunoreactive (irINSL5) neurons in the paraventricular, supraoptic, accessory secretory, and supraoptic retrochiasmatic nuclei, and immunoreactive cell processes in the internal layer of the median eminence.^14^ This is the first evidence that this peptide is expressed in a population of cells in the mouse hypothalamus and pituitary, and that it elevates internal [Ca^2+^] by a mechanism that involves both Ca^2+^ influx and Ca^2+^ release from intracellular stores. The high concentration of irINSL5 in the hypothalamic-pituitary axis suggests a neuroendocrine function of this insulin superfamily member in the mouse.

INSL5 shows the highest sequence similarity to H3 relaxin,^15^ and was predicted to bind to relaxin family peptide (RXFP) receptors. Additionally, both the INSL5 and RXFP4 genes are dysfunctional in the rat and dog genomes; this suggested that INSL5 is the native ligand of RXFP4.^11^ It was subsequently shown that, in vitro, INSL5 binds to RXFP4 with an affinity equal to that of H3 relaxin.^11^ Importantly, INSL5 does not activate RXFP3, however, it does bind to the receptor with low affinity and can act as a weak antagonist. INSL5 neither shows binding affinity for nor activity on either RXFP1 or RXFP2. These results together with their coevolution and partially overlapping tissue expression patterns strongly suggest that INSL5 is an endogenous ligand for RXFP4.^11^

While the primary functions of the INSL5–RXFP4 pair remains unknown, its expression profile, unlike other relaxin family members (e.g., H2 relaxin, INSL3, etc.), suggests a nonreproductive role. In order to systematically investigate its structural and in vitro biological properties, an effective means of producing sufficient quantities of this peptide and its analogues is needed. We present here a significant contribution to INSL5 research, namely the first optimized chemical synthesis protocol for its preparation. We also report here the solution conformation of the peptide and confirm an insulin-like fold. Finally, we characterize its activity on the INSL5 receptor, RXFP4.

## Results and Discussion

### Synthesis of INSL5 A chain

**Figure 2:**
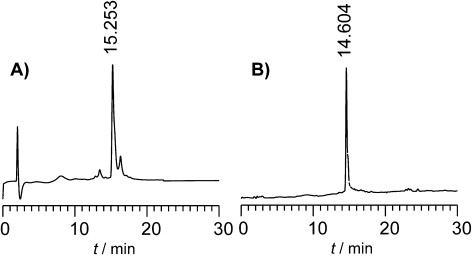
RP-HPLC profile of: A) crude reduced [Cys7,12 (SH), Cys8 (But), Cys21 (Acm)], and B) purified oxidized human [Cys8 (But), Cys21 (Acm)] INSL5 A chain; eluant A: 0.1% aq. TFA; eluant B: 0.1% TFA in acetonitrile; gradient=20–50% B over 30 min; Phenomenex C18 column, pore size 300 Å, particle size 5 μm, 4.6×250 mm.

The INSL5A chain was found to be unusually difficult to synthesize with standard Fmoc-continuous flow methodology with piperidine (20%)/DMF solution for *N*^α^-Fmoc removal and HBTU as a coupling agent. A potential source of the difficulty is the presence of multiple Asp residues. The most frequently encountered side reaction that affects Asp residues during solid phase peptide synthesis (SPPS) is aspartimide formation; this results from a ring closure between the nitrogen of the α-carboxyl amide bond and the β-carboxyl side chain, with the loss of the ester protecting group.^16^ It is a particularly serious problem in Fmoc-SPPS when cyclization is driven by base used to effect Fmoc-group removal.^17^,^18^ The INSL5 A chain was predicted to be more susceptible to aspartimide formation because of the presence of the Asp–Gly sequence in the middle of the chain. It has been previously reported that the Asp–Gly sequence causes as much as 0.5% aspartimide formation per *N*^α^-Fmoc deprotection cycle.^19^ It can be exacerbated by the use of 1,8-diazabicyclo[5.4.0]undec-7-ene (DBU), which has been shown to more effectively promote aspartimide formation than piperidine. Of the various approaches that have been advocated to overcome this problem, such as the addition of dinitrophenol or HOBt to the piperidine solution, only the masking of the Asp–Gly amide bond with 2-hydroxy-4-methoxybenzyl (Hmb) offers complete protection.^19^,^20^ However, the acylation of the Hmb derivatives can be problematic, and the reaction is difficult to follow and often requires nonstandard acylation conditions. One solution is to use the commercially available dipeptide, Fmoc-Asp(O*t*Bu)-(Hmb)Gly-OH.^21^ This derivative is introduced by using standard coupling methods and extends the peptide chain by two residues in one step. Its use has also the additional advantage of overcoming aggregation during chain extension. However, use of this dipeptide is not a cost-effective way to generate peptides. The coupling of the amino acid following this dipeptide is also problematic because of the presence of bulky Hmb side chain in the dipeptide. However, the much cheaper Gly derivative, Fmoc-(Dmb)Gly-OH, which is now commercially available, can also effectively prevent aspartimide formation and chain aggregation.^22^ Use of Fmoc-(Dmb)Gly-OH in the middle of the chain together with one pseudoproline, Fmoc-Leu-Ser(ψ^MeMe^Pro)-OH, at the C-terminal region resulted in a crude A-chain peptide with an excellent HPLC profile (Figure 2).

### Synthesis of INSL5 B chain

**Figure 3:**
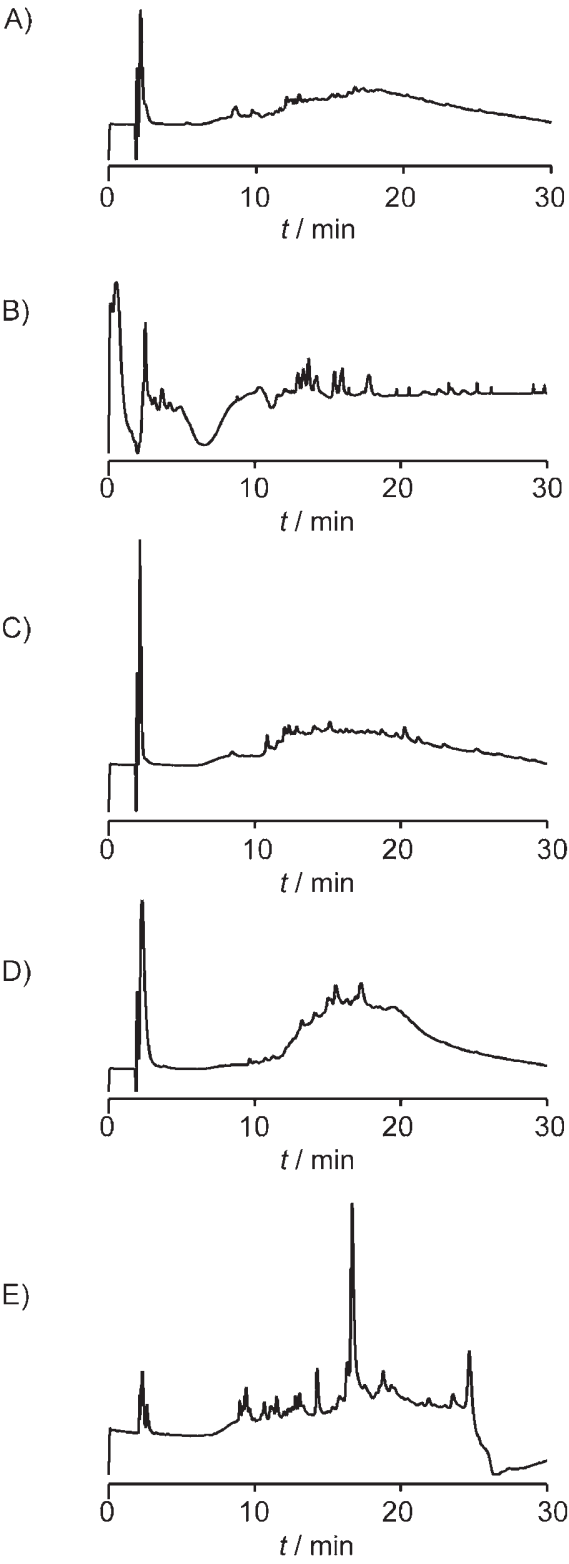
RP-HPLC profile of crude [Cys7 (SH), Cys19 (Acm)] INSL5 B chain obtained under different SPPS conditions: A) synthetic peptide obtained by using standard reaction conditions, that is, Fmoc-deprotection with piperidine (20%), 30 min acylation with HBTU-activated amino acid; B) microwave-assisted acylation and deprotection; C) after introduction of a psuedoproline, Fmoc-Ser(*t*Bu)-Ser(ψ^MeMe^Pro)-OH, into the sequence; D) use of HATU as acylating reagent, reaction time 1 h; E) use of DBU for Fmoc removal; (eluant A: 0.1% aq. TFA; eluant B: 0.1% TFA in acetonitrile; gradient=10–90% B over 30 min; Phenomenex C18 column, pore size 300 Å, particle size 5 μm, 4.6×250 mm).

The B chain of INSL5 was also very difficult to synthesize. The use of standard Fmoc-continuous flow methodology with piperidine (20%)/DMF and HBTU gave a crude product that was shown by both RP-HPLC and MALDI-TOF MS to possess multiple deletion peptides (Figure 3A). As the peptide does not contain an Asp residue, aspartimide formation is not a contributor to the poor quality. During Fmoc-SPPS, aggregation through interchain association of the growing resin-bound peptides is known to contribute to the difficulty of the synthesis.^23^,^24^ Aggregation typically results in a decrease in the rates of acylation and deprotection and consequently leads to the production of deletion products of various length and composition. Such a decrease in the purity of the crude material often results in subsequent difficult purification. Strategies to combat Fmoc-SPPS resin-bound aggregation and improve synthetic product yield include the use of more potent acylation reagents,^25^ backbone amide protection,^26^,^27^ incorporation of pseudoproline,^28^,^29^ microwave irradiation to enhance coupling efficiency,^30^ SPPS at elevated temperatures,^30^ and use of DBU instead of piperidine to deprotect the N-terminal Fmoc group.^31^ A repeat assembly of the INSL5 B chain was undertaken by using microwave-assisted coupling and deprotection. However, it did not afford any improvement in crude peptide purity (Figure 3B). A third assembly of the chain was then undertaken by using a psuedoproline, Fmoc-Ser(*t*Bu)-Ser(ψ^MeMe^Pro)-OH, at the C terminus without success (Figure 3C). A fourth assembly of the peptide was undertaken by using HATU as coupling reagent instead of HBTU and an increase in amino acid acylation time from 30 min to 1 h. Again, no significant improvement in the synthetic product was observed by RP-HPLC (Figure 3D). Finally, use of default conditions (described in the legend of Figure 3A) together with simple replacement of piperidine with the stronger base, DBU (2%)/DMF, which has previously been shown in our laboratory to be effective for Fmoc deprotection in “difficult sequences”,^31^ and no other synthesis protocol change resulted in very good quality crude product, which was shown by MALDI-TOF MS to comprise the correct molecular mass (Figure 3E). This showed that incomplete *N*^α^-Fmoc group removal was, in fact, the major problem in Fmoc-SPPS of the INSL5 B chain rather than incomplete acylation. This is further highlighted by the fact that this simple protocol did not use a pseudoproline residue in the assembly.

### Chain combination

**Figure 4:**
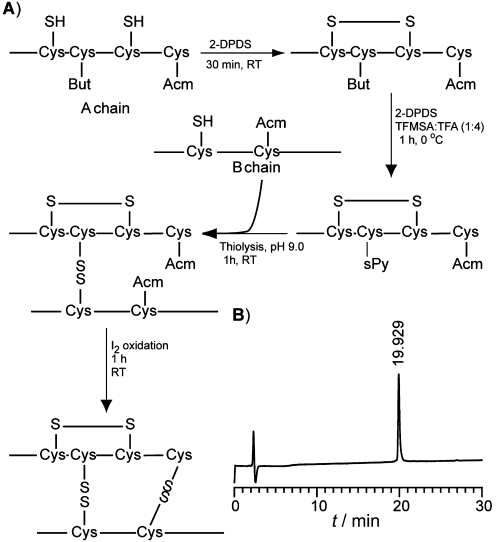
A) Scheme for regioselective disulfide bond formation in human INSL5. B) RP-HPLC profile of the purified synthetic human INSL5 (eluant A: 0.1% aq. TFA; eluant B: 0.1% TFA in acetonitrile; gradient: 20–50% B over 30 min; Phenomenex C18 column, pore size 300 Å, particle size 5 μm, 4.6×250 mm).

Until recently, relaxin and other insulin-like peptides were commonly prepared in our laboratory by random combination of the individual S-reduced A and B chains.^32^–^34^ Following their chemical synthesis, the purified chains are combined in solution at high pH to produce the target peptide in modest to good overall yield. Surprisingly, however, both INSL4 and H3 relaxin have not been successfully prepared by the random-combination method. For this reason, a “forced” technique, that is, regioselective disulfide bond formation was applied to successfully synthesize not only INSL4^35^ and H3 relaxin,^36^ but also equine^37^ and mouse relaxins.^38^ A variety of insulins, relaxins, and their analogues have now been synthesized successfully by using this approach.^39^–^41^ Therefore, to avoid the uncertainty of the random-combination method, this approach has been used here for the synthesis of INSL5. Differential cysteine S-protecting groups (specifically, Trt, But, and Acm) were used to allow their sequential removal or modification followed by directed formation of three disulfide bonds (for details see the Experimental Section). Solid phase synthesis of the separate, selectively S-protected A and B chains followed by their purification and subsequent stepwise formation of each of the three disulfides by oxidation, thiolysis, and iodolysis (Figure 4A) led to the successful acquisition of INSL5 (3.5% overall yield relative to starting B chain) in very high purity as assessed by RP-HPLC (Figure 4B) and MALDI-TOF MS. This yield compares well to that obtained for synthetic H3 relaxin (6%).^36^

### Conformation of the peptide

**Figure 5:**
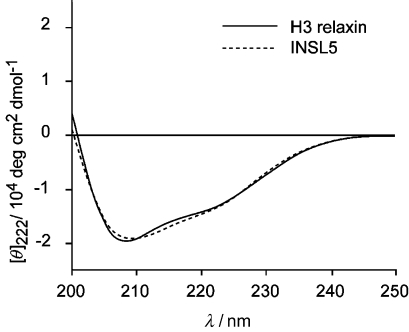
CD spectra of human INSL5 and H3 relaxin in phosphate buffer (10 mm) with NaCl (120 mm, pH 7.4).

*Circular dichroism (CD) spectroscopy*: The conformation of the synthetic human INSL5 was analyzed by CD spectroscopy (Figure 5). The CD for H3 relaxin was also measured for comparison because of its sequence similarity with INSL5. The studies revealed that both peptides possess a significant degree of α-helical conformation along with β sheet and/random-coiled structure. The α-helical content of INSL5, which was calculated from the mean residual weight ellipticity at 222 nm, [*θ*]_222_,^42^ was found to be 38.28% ([*θ*]_222_=13505.0), which is almost the same as H3 relaxin ([*θ*]_222_=13384.0, helix content=37%).

**Figure 6:**
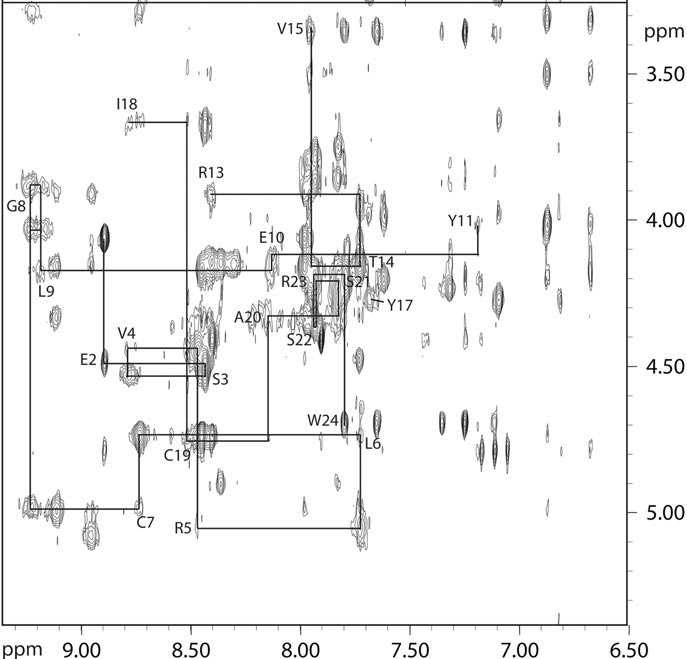
2D NMR spectrum of INSL5. NOESY spectrum of INSL5 (0.4 mg) recorded at pH∼4, 298 K, and 900 MHz. The parts of the “sequential walk” that were used for resonance assignments are marked with connecting lines for resonances in the B chain. The intraresidual Hα–HN cross-peaks are labeled with residue numbers and single letter amino acid codes. The great signal dispersion both in the NH dimension and the Hα dimension is indicative of a well-structured peptide.

*NMR spectroscopy*: To gain further insight into the fold of the peptide and to confirm its insulin-like structure we subjected the peptide to high-field solution NMR spectroscopy. As is evident from Figure 6, which shows the fingerprint region of a NOESY spectrum, the spectral data were of high quality and showed excellent signal dispersion; this is consistent with a highly structured peptide. Partial resonance assignments were achieved by using 2D sequential assignment strategies and the assignments and secondary chemical shifts were consistent with the insulin-like fold, as reported in the NMR spectroscopy studies of the H3 relaxin and INSL3 peptides.^43^,^44^ The fold is characterized by mainly helical regions, but a small extended region with cross-strand interactions between the two peptide chains is also present.

### Activity on RXFP3 and RXFP4

**Figure 7:**
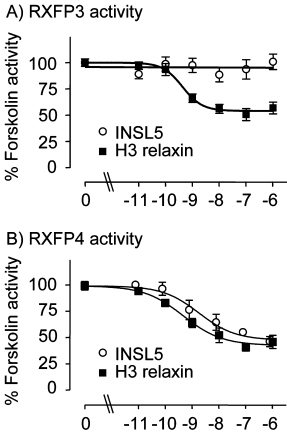
The cAMP activity of human INSL5 compared to H3 relaxin in RXFP3 and RXFP4 transfected cells. Data are mean±SEM of triplicate determinations from at least four independent experiments.

The synthetic INSL5 peptide was tested for its ability to inhibit forskolin induced cAMP activity in RXFP3 and RXFP4 transfected cells (Figure 7). As expected the peptide did not demonstrate any activity on RXFP3, although H3 relaxin displayed similar high activity to that previously reported^36^ (Table 1). In contrast INSL5 was able to decrease forskolin induced cAMP activity in RXFP4 transfected cells in a dose-dependent manner. The activity of the peptide was slightly lower than H3 relaxin (Table 1) but similar to that previously described.^11^

**Table 1:** Activity (pEC_50_[a]) of INSL5 and H3 relaxin on RXFP3 and RXFP4.

Peptide	RXFP3 pEC_50_	RXFP4 pEC_50_
INSL5	n.a.[b]	8.82±0.24[c] (*n*=5)
H3 relaxin	9.37±0.18 (*n*=5)	9.43±0.16 (*n*=4)

[a] The pEC_50_ is defined as the negative logarithm of EC_50_—the concentration of agonist (INSL5) that produced 50% maximal response;

[b] no activity;

[c] *p*<0.01 versus H3 relaxin.

## Conclusions

We have described a highly efficient protocol through which human INSL5 was successfully prepared for the first time in good overall yield by a combination of SPPS and regioselective disulfide bond formation. The synthetic INSL5 shows a typical insulin-like α-helical content along with β sheet and/random-coiled structure according to CD spectroscopy, and, furthermore, has an overall similar structure to H3 relaxin—a related member of the insulin superfamily—based on NMR spectroscopy data. The peptide was able to inhibit cAMP activity in SK-N-MC cells that expressed the human RXFP4 receptor with a similar activity to H3 relaxin. In contrast, it had no activity on the human RXFP3 receptor. Synthetic human INSL5, therefore, demonstrates equivalent activity to the recombinant peptide and will be a valuable probe for testing its biological function.

## Experimental Section

**General**: Fmoc amino acid derivatives for peptide synthesis were of the l configuration and were purchased from Auspep Pty. Ltd. (Melbourne, Australia) or GL Biochem (Shanghai, China). RP-HPLC columns were obtained from Phenomenex (Torrance, California, USA). Solvents and chemicals were all peptide synthesis or analytical grade.

**SPPS**: The predicted primary structure of INSL5 consists of a twenty one residue A chain and a twenty four residue B chain that are linked by three disulfide bonds (Figure 1). Both regioselectively S-protected A and B chains were separately synthesized by using the continuous flow Fmoc solid-phase method^45^ with an automatic PerSeptive Biosystems Pioneer peptide synthesizer (Farmingham, MA, USA), as previously reported.^46^ The solid support used for both A and B chains was Fmoc-PAL-PEG-PS and a fourfold molar excess of HBTU-activated l-Fmoc-amino acids were used throughout. All amino acid side chains were protected by trifluoroacetic acid (TFA)-labile protecting groups except for Cys8 (But) and Cys21 (Acm) in the A chain, and Cys19 (Acm) in the B chain. The scale of assembly was 0.1 mmol for each of the two chains. The acylation (coupling) reaction was carried out between 30 min to 1 h. Deprotection of the Fmoc group was with piperidine (20%)/DMF or DBU (2%)/DMF. At the end of the synthesis, cleavage from the solid supports and side-chain deprotection was achieved by 2 h treatment with TFA (94%) in the presence of scavengers: anisole (3%), 3,6-dioxa-1,8-octanedithiol (DODT, 2%),^47^ and triisopropylsilane (TIPS, 1%). The identities of the crude peptides were confirmed by MALDI-TOF MS.

**A chain intramolecular disulfide bond formation**: The crude [Cys8 (But), Cys21 (Acm)] A chain (190 mg, 81.55 μmol) was dissolved in GnHCl (6 m, 100 mL) with Gly⋅NaOH (0.1 m, pH 9) and diluted with water (500 mL); subsequently 2-pyridyl disulfide (DPDS; 57 mL of a 1 mm solution in MeOH) was added.^48^ The reaction progress was monitored by analytical RP-HPLC. The retention time of the intramolecularly oxidized A chain was earlier than that of the reduced A chain (Figure 2). The reaction was completed in 30 min at room temperature. The large volume of solution was loaded onto the preparative column through the pump’s C-line and purified in several runs. The combined product was identified by MALDI-TOF MS; *m*/*z* 2331.488 [*M*+H]^+^, calcd 2330.59. The purified solution was freeze dried to give 60.0 mg of purified [Cys8 (But), Cys21 (Acm)] A chain (yield 31.6%).

**A-chain conversion of Cys8 (But) to Cys8 (Pyr)**: The purified [Cys8 (But), Cys21 (Acm)] A chain (58.6 mg, 25.2 μmol) and 2-DPDS (22.0 mg, 100.0 μmol) were dissolved in TFA/anisole (9:1, *v*/*v*; 1.63 mL). The solution was chilled on ice before TFMSA/TFA (1:4, *v*/*v*; 1.63 mL) was added. The reaction was performed for 1 h at 0°C, and the peptide was collected by diethyl ether precipitation, centrifuged, and purified by preparative RP-HPLC. The freeze dried purified product weighed 21.0 mg (8.81 μmol, 31.2%) and was identified by MALDI-TOF MS; *m*/*z* 2384.15 [*M*+H]^+^, calcd 2384.59.

**Chain combination**: The [Cys8 (Pyr), Cys21 (Acm)] A chain peptide (20.0 mg, 8.4 μmol) was dissolved in GnHCl (6m), Gly⋅NaOH buffer (0.1m, pH 9; 10 mL). The [Cys7 (S-thiol), Cys19 (Acm)] B chain (25.0 mg, 8.6 μmol) was dissolved in deionized water (6 mL) and was added slowly to the A chain solution. The reaction was monitored by analytical RP-HPLC and the product was identified by MALDI-TOF MS; *m*/*z* 5187.45 [*M*+H]^+^, calcd 5187.57. The reaction was complete within 1 h at room temperature under a nitrogen atmosphere. Neat TFA was added and the product was purified by preparative RP-HPLC and freeze dried to give 15.50 mg (2.98 μmol, 34.7% relative to B chain) of [Cys21A (Acm)/Cys19B (Acm)]–A–B.

**INSL5**: The A–B peptide [Cys21A (Acm)/Cys19B (Acm)] (14.0 mg, 2.7 μmol) was dissolved in glacial acetic acid (8.5 mL) and HCl (60 mm, 1.0 mL), and iodine/acetic acid (11.4 mL of a 20 mm solution) was then added to the solution. The reaction was monitored by analytical HPLC. After 1 h the reaction was stopped by direct addition of ice-cold diethyl ether. The final product was purified with preparative HPLC, and freeze dried to give 1.4 mg (0.278 μmol, 10.3%). The peptide was identified by MALDI-TOF MS as a single species; *m*/*z* 5043.721 [*M*+H]^+^, calcd 5043.015; the purity was examined by analytical RP-HPLC (Figure 4). The amino acid composition and peptide content (38.8%) was determined by amino acid analysis.

**Circular dichroism spectroscopy**: CD spectra were recorded by using JASCO (J-185, Tokyo, Japan) at room temperature (20°C) with a 1 mm path length cell. The peptides were dissolved in phosphate buffer (10 mm) with NaCl (120 mm, pH 7.4) at a concentration of 0.1 mgmL^−1^.

**NMR spectroscopy**: For NMR spectroscopy a sample that contained INSL5 (1 mg in 0.5 mL 90% H_2_O/10% D_2_O) was prepared. The 2D homonuclear spectra, including TOCSY and NOESY, with a mixing time of 150 ms spectra were recorded at pH∼4 and 298 K by using a Bruker Avance 900 MHz spectrometer and processed with Topsin (Bruker).

**cAMP activity assays**: The synthetic INSL5 peptide was tested for its ability to inhibit cAMP activity in SK-N-MC cells (human caucasian neuroblastoma cells) that transiently expressed either RXFP3 or RXFP4. Human RXFP3 and RXFP4 in the mammalian expression vector pcDNA3.1(+)zeo were obtained from the UMR cDNA Resource Center (http://www.cdna.org). The SK-N-MC cells were cultured in a 1:1 mixture of Dulbecco’s modified Eagle’s medium (DMEM; Multicel) and HAMS-F12 (Multicel) supplemented with fetal bovine serum (10%) and incubated in a humidified incubator with 5% CO_2_ at 37°C. Cells were seeded at 30000 cells per well in 96-well plates that were coated with poly-l-lysine (Sigma), and were incubated, overnight, at 37°C. After incubation they were transfected with the receptor of interest together with a pCRE β-galactosidase reporter plasmid^49^ and then incubated for a further 24 h. The cAMP activity of INSL5 in comparison to H3 relaxin was then assessed in cells stimulated with forskolin (5 μm) as previously described.^50^ Peptides were measured in triplicate within each assay and each experiment was repeated at least four times. Data were plotted by using GraphPad Prism 4 and statistical differences in pEC50 values were determined with a Student t-test.
